# Ceramide Synthase 2 Null Mice Are Protected from Ovalbumin-Induced Asthma with Higher T Cell Receptor Signal Strength in CD4+ T Cells

**DOI:** 10.3390/ijms22052713

**Published:** 2021-03-08

**Authors:** Sun-Hye Shin, Kyung-Ah Cho, Hee-Soo Yoon, So-Yeon Kim, Hee-Yeon Kim, Yael Pewzner-Jung, Sung-Ae Jung, Woo-Jae Park, Anthony H. Futerman, Joo-Won Park

**Affiliations:** 1 Department of Biochemistry, College of Medicine, Ewha Womans University, Seoul 07804, Korea; s1sunhye@naver.com (S.-H.S.); gltn129@naver.com (H.-S.Y.); hithdus1@naver.com (S.-Y.K.); heeyeon1432@gmail.com (H.-Y.K.); 2 Department of Microbiology, College of Medicine, Ewha Womans University, Seoul 07804, Korea; kyungahcho@ewha.ac.kr; 3 Department of Biomolecular Sciences, Weizmann Institute of Science, Rehovot 76100, Israel; yael.pewzner-jung@weizmann.ac.il (Y.P.-J.); tony.futerman@weizmann.ac.il (A.H.F.); 4 Department of Internal Medicine, College of Medicine, Ewha Womans University, Seoul 07804, Korea; jassa@ewha.ac.kr; 5 Department of Biochemistry, College of Medicine, Gachon University, Incheon 21999, Korea

**Keywords:** asthma, ceramide, chain length, ceramide synthase 2, T cell receptor strength

## Abstract

(1) Background: six mammalian ceramide synthases (CerS1–6) determine the acyl chain length of sphingolipids (SLs). Although ceramide levels are increased in murine allergic asthma models and in asthmatic patients, the precise role of SLs with specific chain lengths is still unclear. The role of CerS2, which mainly synthesizes C22–C24 ceramides, was investigated in immune responses elicited by airway inflammation using CerS2 null mice. (2) Methods: asthma was induced in wild type (WT) and CerS2 null mice with ovalbumin (OVA), and inflammatory cytokines and CD4 (cluster of differentiation 4)+ T helper (Th) cell profiles were analyzed. We also compared the functional capacity of CD4+ T cells isolated from WT and CerS2 null mice. (3) Results: CerS2 null mice exhibited milder symptoms and lower Th2 responses than WT mice after OVA exposure. CerS2 null CD4+ T cells showed impaired Th2 and increased Th17 responses with concomitant higher T cell receptor (TCR) signal strength after TCR stimulation. Notably, increased Th17 responses of CerS2 null CD4+ T cells appeared only in TCR-mediated, but not in TCR-independent, treatment. (4) Conclusions: altered Th2/Th17 immune response with higher TCR signal strength was observed in CerS2 null CD4+ T cells upon TCR stimulation. CerS2 and very-long chain SLs may be therapeutic targets for Th2-related diseases such as asthma.

## 1. Introduction

Asthma is a multi-faceted disease characterized by chronic airway inflammation and variable remodeling, which results in a range of clinical presentations, such as wheezing, coughing, shortness of breath, chest tightness, bronchial hyper-responsiveness, and airflow limitation [1]. Asthma can substantially impact the quality of life, and asthma is globally ranked 16th among the leading causes of years lived with disabilities, and 28th among the leading causes of disease burdens, as measured by disability-adjusted life years [2]. Asthma affects approximately 300 million people worldwide, and it is estimated that a further 100 million may be affected by 2025 [2]. Airway inflammation is a prominent feature of asthma, and type 2 immune response-associated inflammation occurs in >80% of children and in the majority of adults with asthma caused by sensitization to environmental allergens [1].

The type 2 immune response directs distinct immune responses that are mainly regulated by subpopulations of CD4 (cluster of differentiation 4)+ T cells, known as T helper (Th) 2 cells [3]. Naïve CD4+ T cells are activated by recognition of antigens, which are presented on the class II major histocompatibility complex in antigen-presenting cells, via an interaction with the T cell receptor (TCR) [4]. Upon activation, naïve CD4+ T cells can differentiate into a clone of effector cells, which can be divided into three major types, referred to as Th1, Th2, and Th17 cells, with distinct cytokine-secretion phenotypes [4,5]. The phenotype of a polarized T cell that differentiates from a naïve precursor is determined by the complex interaction of antigen-presenting cells with naïve T cells, and involves a multitude of factors, including a dominant cytokine environment, co-stimulatory molecules, the types and loads of antigens presented, and a plethora of signaling cascades [4]. Th1 cells mainly mediate cellular immune responses by producing the cytokine, interferon-γ (IFN-γ), and Th2 cells potentiate a humoral response by secreting interleukin (IL)-4, -5, -10, and -13, which upregulate antibody production [4]. Th17 cells mainly secrete cytokines such as IL-17A, IL-17F, IL-6, and IL-22, and have been implicated in the pathogenesis of many inflammatory and autoimmune diseases [4,6]. Although recent advances have revealed the association of Th1 and Th17 cells with severe, steroid-resistant asthma, which is often marked by neutrophil infiltration, Th2 cells promote eosinophil recruitment, and are classically considered a predominant contributor to the development of asthma [4].

Sphingolipids (SLs) are a class of lipids with a long chain base, normally sphingosine (d18:1) or sphinganine (d18:0), and exhibit high structural diversity and bioactive properties. The roles of SLs in various biological functions have not been fully elucidated. De novo synthesis of SLs begins with condensation of serine and palmitoyl-CoA by serine-palmitoyltransferase, which generates 3-ketosphinganine [7]. Moreover, 3-ketosphinganine is then transformed to sphinganine by 3-ketosphinganine reductase [8], and sphinganine is converted to dihydroceramide via an N-acylation process by ceramide synthases (CerS) [9]. Next, dihydroceramide desaturase introduces the 4,5-*trans* double bond into dihydroceramide, generating ceramide [10,11]. Ceramide is regarded as a central hub of SL metabolism, due to its critical role in SL accumulation through de novo synthesis, as well as serving as a precursor for other SL metabolites, such as sphingosine, glycosphingolipids, ceramide-1-phosphate, and sphingomyelin [12]. CerS determine the acyl chain length of ceramide, with six CerS existing in mammals [9]. CerS1 preferentially generates C18-ceramide, and CerS2 generates C22–C24 ceramides, also known as very-long-chain-ceramides [13,14]. CerS3 and CerS4 mainly produce C26–C34 ceramides and C18–C20 ceramides, respectively [15,16]. CerS5 and CerS6 primarily mediate the synthesis of C16-ceramide [17,18]. The distinct roles of ceramides, depending on the acyl chain length, have been recently uncovered [8,19]. For example, the acyl chain length of SLs exert different effects on endoplasmic reticulum stress [11], biophysical properties of membranes [20], cancer cell growth [21], and regulation of cell death [8]. Importantly, CerS2 and CerS6 have opposing effects in radiation-induced apoptosis and endoplasmic reticulum stress [22,23]. Similarly, while CerS2 null mice are protected from experimental autoimmune encephalomyelitis, CerS6 deficient mice show aggravated phenotypes in the same model [24,25].

Recently, the roles of CerS6-generated ceramides in modulating T cell activation and function were reported, and CerS6 was required for efficient TCR signal transduction [26]. Considering the distinct roles of SLs, depending on their acyl chain lengths, the effects of CerS2 on CD4+ T cell activation and differentiation were investigated using CerS2 null mice [27]. Decreased Th2 and increased Th17 response with higher TCR signal strength was observed in CerS2 null CD4+ T cells upon TCR stimulation and the severity of ovalbumin (OVA)-induced asthma was decreased in CerS2 null mice, implicating a pathological role of very-long-acyl chain SLs in asthma.

## 2. Results

### 2.1. CerS2 Null Mice Show Milder Phenotypes in OVA-Induced Airway Inflammation Compared to Wild Type Mice

**Figure 1:**
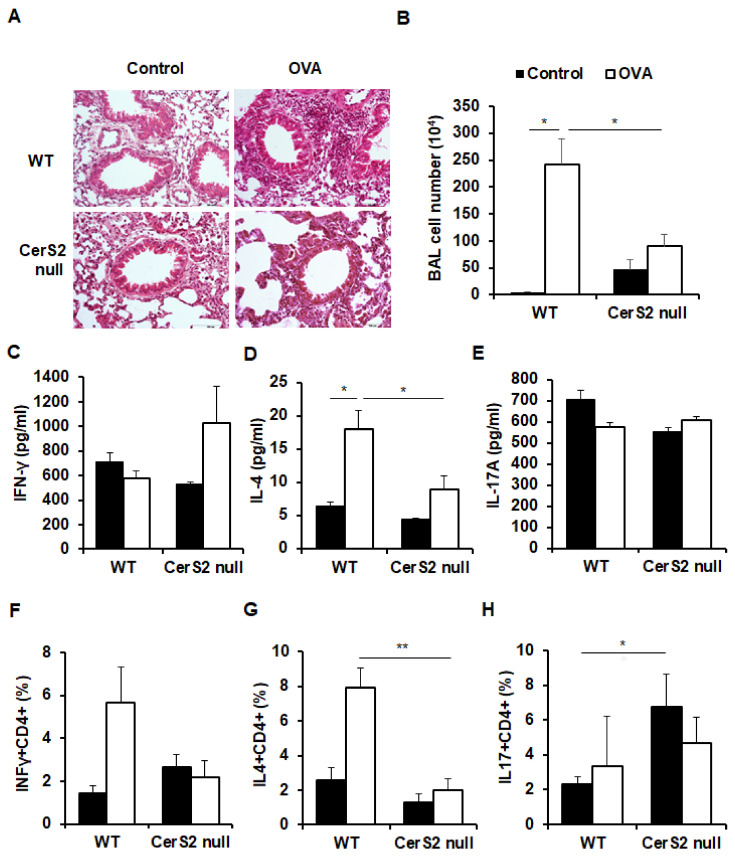
Wild type (WT) and ceramide synthase 2 (CerS2) null mice exhibit different immune responses in airway inflammation. (**A**) Paraffin-embedded lung sections were prepared 24 h after the last ovalbumin (OVA) or phosphate-buffered saline (PBS) challenge, then stained with hematoxylin and eosin to visualize inflammation (magnification: ×200). (**B**) Bronchoalveolar lavage (BAL) fluid was collected 24 h after the final ovalbumin or PBS challenge, and the number of cells in the BAL fluid was determined. Protein levels of (**C**) interferon-γ (IFN-γ), (**D**) interleukin (IL)-4, and (**E**) IL-17 in the BAL fluid were measured using ELISA. Helper T (Th) cell profiles were compared by measuring (**F**) CD4 (cluster of differentiation 4)+ IFN-γ+ Th1 cells, (**G**) CD4+ IL-4+ Th2 cells, and (**H**) CD4+ IL-17+ Th17 cells using flow cytometric analyses. Data are means ± SEM (*n* = 6). * *p* < 0.05; ** *p* < 0.01. Statistical significance was analyzed using the two-way analysis of variance.

An OVA sensitization protocol was used to induce allergic airway inflammation in wild type (WT) and CerS2 null mice [28]. Twenty-four hours after the last challenge via the inhalation of 1% OVA aerosol solution, mice were subjected to bronchoalveolar lavage (BAL). Histological observation of OVA-challenged WT and CerS2 null mice revealed cellular infiltration in the lungs; however, WT mice showed higher degree of immune cell infiltration compared with CerS2 null mice (Figure 1A). This was also confirmed by analysis of BAL cell numbers (Figure 1B). Next, we compared the type of immune response elicited by OVA challenge by measuring levels of Th1 IFN-γ, Th2 IL-4, and Th17 IL-17 in BAL fluid (Figure 1C–E). After OVA treatment, ELISA analysis showed no significant difference between WT and CerS2 null mice in levels of IFN-γ and IL-17 (Figure 1C,E). However, CerS2 null mice exhibited a significantly decreased level of IL-4 compared with WT mice during OVA-induced airway inflammation (Figure 1D). Next, we determined intracellular expression levels of IFN-γ, IL-4, and IL-17 between CD4+ BAL cells using flow cytometry (Figure 1F–H and Figure S1A–C). Similar to the protein levels of IL-4 in BAL fluid, the levels of CD4+ IL-4+ cells were highly increased in OVA-challenged WT mice, but not in OVA-challenged CerS2 null mice (Figure 1G). The higher levels of CD4+ IFN-γ+ cells was also shown in OVA-challenged WT mice, but not OVA-challenged CerS2 null mice, although the differences were not statistically significant (Figure 1F). Notably, the basal levels of CD4+ IL-17+ cells were significantly higher in CerS2 null mice compared to WT mice even without OVA challenge (Figure 1H). Together, these results indicated that airway inflammation using OVA aerosol challenge induced significant Th2 responses in WT mice, while CerS2 null mice displayed a lower Th2 response with higher basal levels of Th17 cells in the lungs.

### 2.2. CerS2 Null CD4+ T Cells Proliferate Normally during Stimulation, and CerS2 Expression Positively Correlates with T Cell Activation

**Figure 2:**
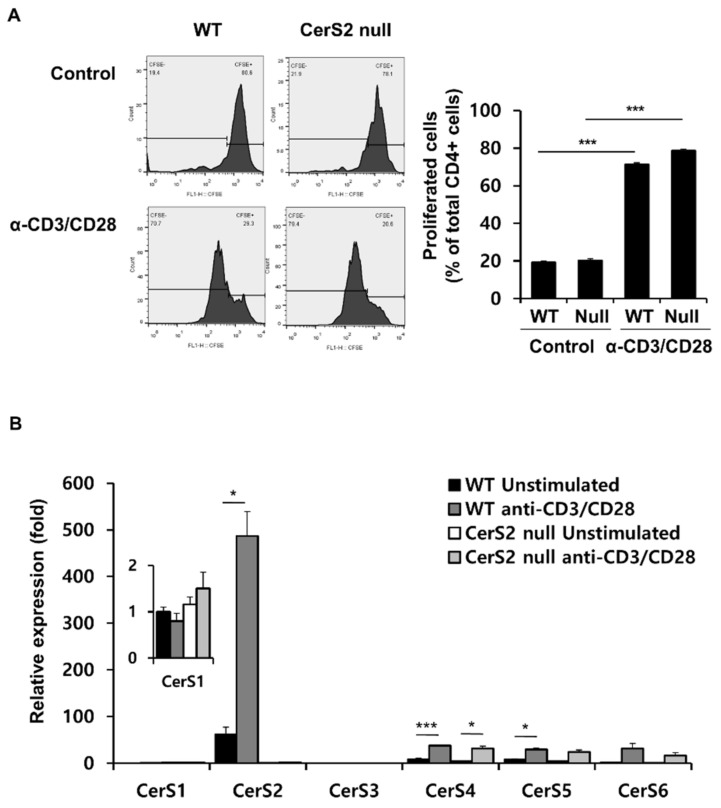
WT-CD4+T and CerS2 null-CD4+T cells show similar proliferation capacities, and CerS2 positively correlates with T cell activation. (**A**) CFSE-labeled CD4+ T cells from wild type and CerS2 null mice were cultured with or without anti-CD3/anti-CD28 antibodies (α-CD3/CD28) for 5 days, and the degree of proliferation was measured using flow cytometry (left). The percentages of proliferating cells was analyzed (right). Data are expressed as means ± SEM (*** *p* < 0.001). (**B**) The mRNA expression of CerS1-6 in T cells cultured with or without anti-CD3/anti-CD28 antibodies for 3 days was confirmed by quantitative real-time PCR. Data are expressed as the mean ± SEM (*n* = 4). * *p* < 0.05; *** *p* < 0.001. Null indicates CerS2 null mice. Statistical significance was analyzed using the two-way analysis of variance.

We examined the proliferation of CD4+ T cells from WT and CerS2 null mice to confirm whether the differential cytokine profiles observed in OVA-challenged mice were due to cell proliferation. CFSE-labeled splenic CD4+ T cells from WT and CerS2 null mice were incubated with anti-CD3 and anti-CD28 antibodies, followed by flow cytometric analyses. Figure 2A shows that splenic CD4 T cells from both WT and CerS2 null mice actively proliferated in response to stimulation using anti-CD3 and anti-CD28 antibodies. The proliferation rates of CD4+ T cells from WT and CerS2 null mice appeared similar (Figure 2A). In addition, the extent of proliferation of CerS2 null-CD4+ T cells was similar with WT upon OVA stimulation (Figure S2). These results indicate that the low production of IL-4 in OVA-CerS2 null mice was not due to low proliferation of T cells. We examined whether CerS expression correlated with T cell activation. When we compared the mRNA expressions of CerS, WT-CD4+ T cells expressed significantly increased levels of CerS2, CerS4, and CerS5 when stimulated with anti-CD3 and anti-CD28 antibodies. In particular, CerS2 was the highest CerS gene in activated CD4+ T cells. CD4+ T cells from CerS2 null mice showed increased levels of CerS4 when stimulated, but lower than those of WT mice (Figure 2B). CerS2 was therefore assumed to positively correlate with T cell activation, even though CerS2 itself did not affect the proliferation capacity of T cells. CerS2 induction by proliferated T cells implied that CerS2 possibly affected further T cell activity, by either specific cellular polarization into specialized Th cells or other mechanisms.

### 2.3. WT and CerS2 Null-CD4+ T Cells Differ in Transcription Factor Expression Involved in Induction of IL-4

**Figure 3:**
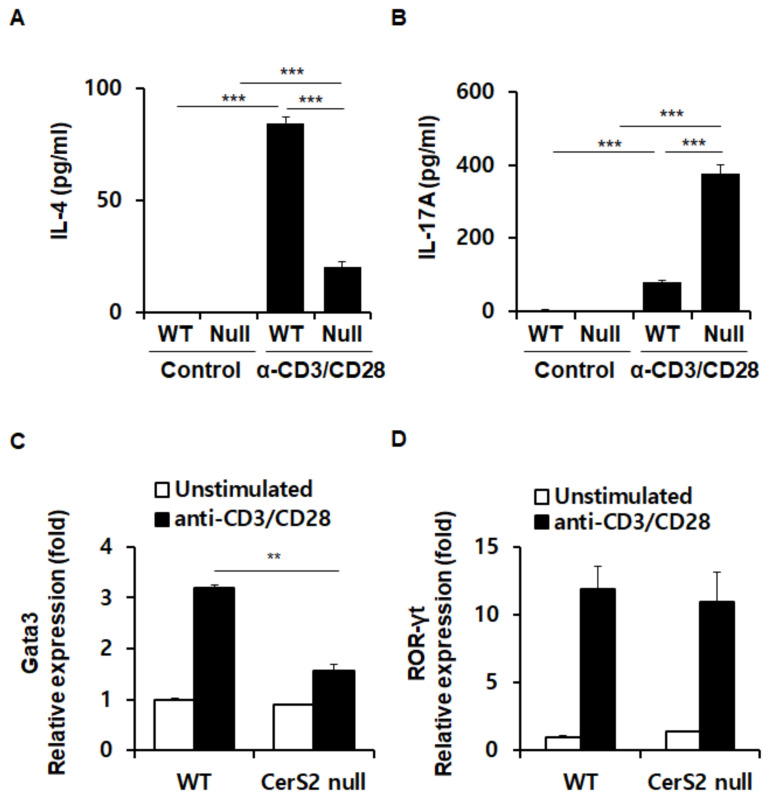
WT-CD4+T cells and CerS2 null-CD4+T cells differ in induction of cytokines and corresponding transcription factors. Splenic CD4+ T cells isolated from wild type and CerS2 null mice were cultured in the presence or absence of anti-CD3/anti-CD28 antibodies (α-CD3/CD28) for 3 days, and the protein levels of (**A**) IL-4, and (**B**) IL-17 in cell culture supernatants were analyzed by ELISA. mRNA expression of (**C**) Gata3, the Th2 cells polarizing transcription factor, and (**D**) RORγt, the Th17 cell polarizing transcription factor, were measured using quantitative real-time PCR. Data are expressed as the mean ± SEM (*n* = 5). ** *p* < 0.01; *** *p* < 0.001. Null indicates CerS2 null mice. Statistical significance was analyzed using the two-way analysis of variance.

Because Th2 and Th17 responses were altered in OVA-challenged CerS2 null mice, we next measured the secretory capacities of cytokines, particularly Th2 cytokine IL-4 and Th17 cytokine IL-17 between CD4+ T cells from WT and CerS2 null mice. Cell culture supernatants collected from splenic CD4+ T cells cultured with or without anti-CD3/anti-CD28 antibodies showed distinct results of IL-4 and IL-17 levels between WT and CerS2 null mice. CerS2 null-CD4+ T cells showed significantly lower secretion of IL-4 compared with those of WT mice (Figure 3A), whereas TCR-stimulated CerS2 null-CD4+ T cells produced significantly higher amounts of IL-17 compared with that of WT-CD4+ T cells (Figure 3B). We then confirmed the cellular mRNA expressions of Gata3 and RAR-related orphan receptor gamma (RORγt), which are master regulators of Th2 and Th17 differentiation, respectively. The expression of Gata3 was induced by stimulation with anti-CD3 and anti-CD28 antibodies in T cells from both WT and CerS2 null mice, but this expression was significantly weaker in CerS2 null-CD4+ T cells (Figure 3C). Similar with Gata3 expression, induction of IL-4 mRNA expression by stimulation with anti-CD3 and anti-CD28 antibodies was lower in CerS2 null-CD4+ T cells than WT cells (data not shown). Notably, RORγt was similarly increased after stimulation in both WT-CD4+ T cells and CerS2 null- CD4+ T cells. Together, these results indicate that differential secretion of IL-17 was due to a regulatory mechanism other than polarizing transcription factor activity.

### 2.4. Helper T (Th) Cells Derived from WT-Naïve CD4+ T Cells and CerS2 Null-Naïve CD4+ T Cells Differentially Produce Signature Cytokines

**Figure 4:**
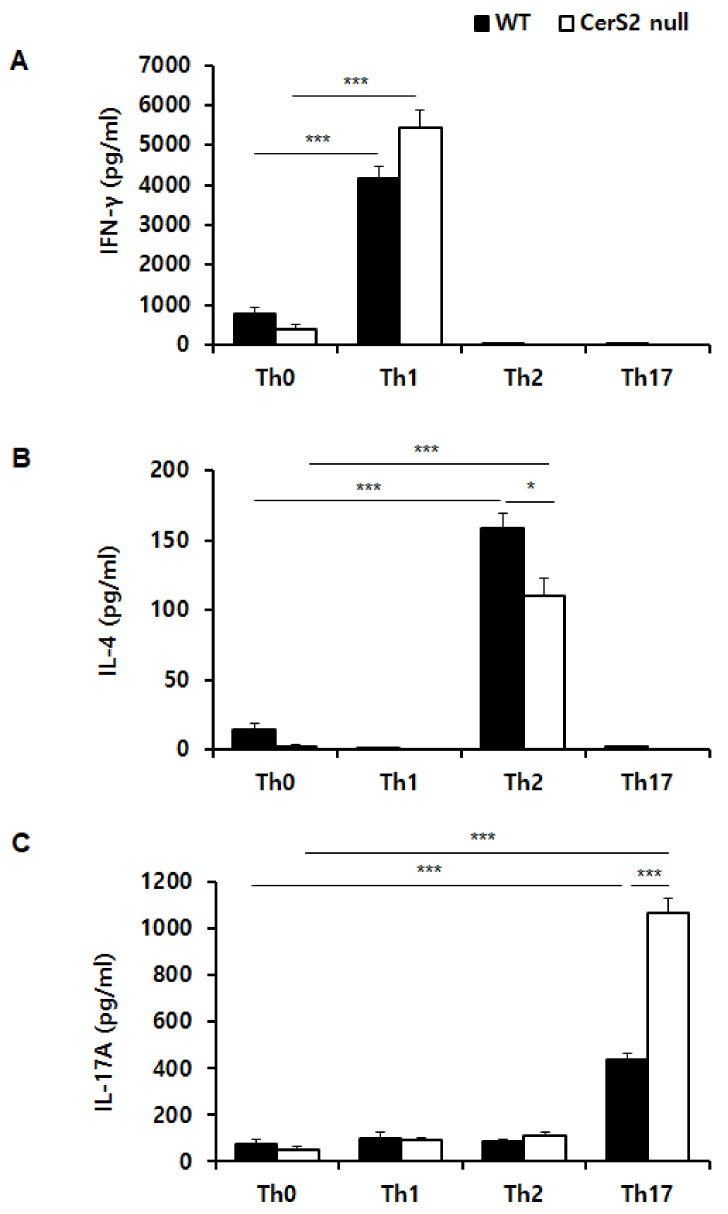
Helper T (Th) cells differentiate from wild type (WT)-naïve CD4+ T cells, and CerS2 null-naïve CD4+ T cells differ in cytokine secretion. Splenic naïve CD4+ T cells from WT and CerS2 null mice were differentiated into Th1, Th2, and Th17, and signature cytokines in cell culture supernatants were measured by ELISA. (**A**) WT-naïve and CerS2 null-naïve CD4+ T cells were differentiated into Th1 cells during Th1 selection conditions and exclusively produced IFN-γ. (**B**) WT-naïve and CerS2 null-naïve CD4+ T cells that differentiated into Th2 cells actively produced IL-4. CerS2 null-Th2 cells secreted significantly lower amounts of IL-4 compared with WT-Th2 cells. (**C**) WT-naïve and CerS2 null-naïve CD4+ T cells successfully produced IL-17 during Th17 polarizing stimulation. CerS2 null-Th17 cells showed significantly higher levels of IL-17 than WT-Th17 cells. Data are expressed as the mean ± SEM (*n* = 4). * *p* < 0.05; *** *p* < 0.001. Statistical significance was analyzed using the two-way analysis of variance.

Because IL-4 and IL-17 cytokine secretion was altered in undifferentiated total CD4+ T cells of CerS2 null mice, we subsequently tested the efficacies of cytokine production in CD4+ T cells, which were differentiated into specific subsets. Figure 4A shows that both WT-naïve CD4+ T cells and CerS2 null-naïve CD 4+ T cells that were differentiated into Th1 cells successfully produced IFN-γ, and that there was no significant difference between WT and CerS2 null mice. However, CerS2 null mice-naïve CD4+ T cells secreted significantly lower amounts of IL-4 than those of WT mice during Th2 selection conditions (Figure 4B). Naïve CD4+ T cells from both WT and CerS2 null mice were differentiated into Th17 cells as shown by a significant increase of IL-17, but Th17 cells from CerS2 null mice produced much higher amounts of IL-17 (Figure 4C).

### 2.5. Differential Secretion of IL-4 and IL-17 between T Cells from WT and CerS2 Null Mice Correlates with TCR Stimulation

**Figure 5:**
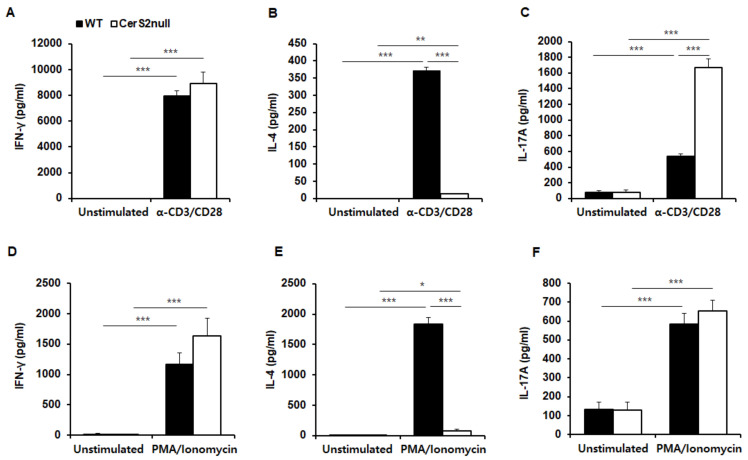
Wild type (WT)-CD4+ T and CerS2 null-CD4+ T cells are stimulated via a T cell receptor (TCR)-dependent or independent manner for cytokine production. Splenic CD4+ T cells from WT and CerS2 null mice were cultured in the presence or absence of anti-CD3/anti-CD28 antibodies (α-CD3/CD28) for 3 days, followed by ELISA to measure secreted levels of (**A**) IFN-γ, (**B**) IL-4, and (**C**) IL-17. For T cell receptor-independent stimulation, splenic CD4+ T cells from WT and CerS2 null mice were cultured in the presence or absence of phorbol 12-myristate 13-acetate (PMA)/ionomycin for 16 h, and the secreted (**D**) IFN-γ, (**E**) IL-4, and (**F**) IL-17 were measured by ELISAs. Data are expressed as the mean ± SEM (*n* = 4). * *p* < 0.05; ** *p* < 0.01; *** *p* < 0.001. Statistical significance was analyzed using the two-way analysis of variance.

Based on the above results, we investigated whether TCR stimulation was a critical mediator resulting in differences of IL-4 and IL-17 between WT-CD4+ T cells and CerS2 null-CD4+ T cells. Thus, we compared cytokine production in CD4+ T cells under distinct stimulus conditions. Based on ELISA analyses, direct TCR stimulation with anti-CD3 and anti-CD28 antibodies effectively induced IFN-γ, IL-4, and IL-17 in T cells from both WT and CerS2 null mice (Figure 5A–C). Again, the level of IL-4 in CerS2 null-CD4+ T cells was significantly lower than that of WT mice (Figure 5B). Instead, CerS2 null-CD4+ T cells produced significantly higher amounts of IL-17, when compared with WT-CD4+ T cells (Figure 5C). We then stimulated T cells from both mice with phorbol 12-myristate 13-acetate (PMA) and ionomycin, and measured IFN-γ, IL-4, and IL-17 levels. PMA activates protein kinase C, while ionomycin is a calcium ionophore, and stimulation with these compounds bypasses the T cell membrane receptor complex. PMA and ionomycin were used to mimic a TCR-independent stimulus to discriminate TCR-dependent stimulation by anti-CD3 and anti-CD28 antibodies. When stimulated with PMA/ionomycin, T cells from both WT and CerS2 null mice effectively produced IFN-γ with no significant difference, but the levels of IL-4 were significantly lower in CerS2 null-CD4+ T cells (Figure 5D,E). These results were the same as those of T cells during TCR stimulation using anti-CD3 and anti-CD28 antibodies. Notably, IL-17 was similarly induced in T cells from both WT and CerS2 null mice in the presence of PMA/ionomycin (Figure 5F). Because CerS2 null-CD4+ T cells secreted higher levels of IL-17, when the TCR was stimulated by anti-CD3 and anti-CD28 antibodies, these results indicate that the TCR signal may have contributed the higher Th17 response in CerS2 null mice.

### 2.6. WT-CD4+ T Cells and CerS2 Null-CD4+ T Cells Differ in TCR Signal Strength

**Figure 6:**
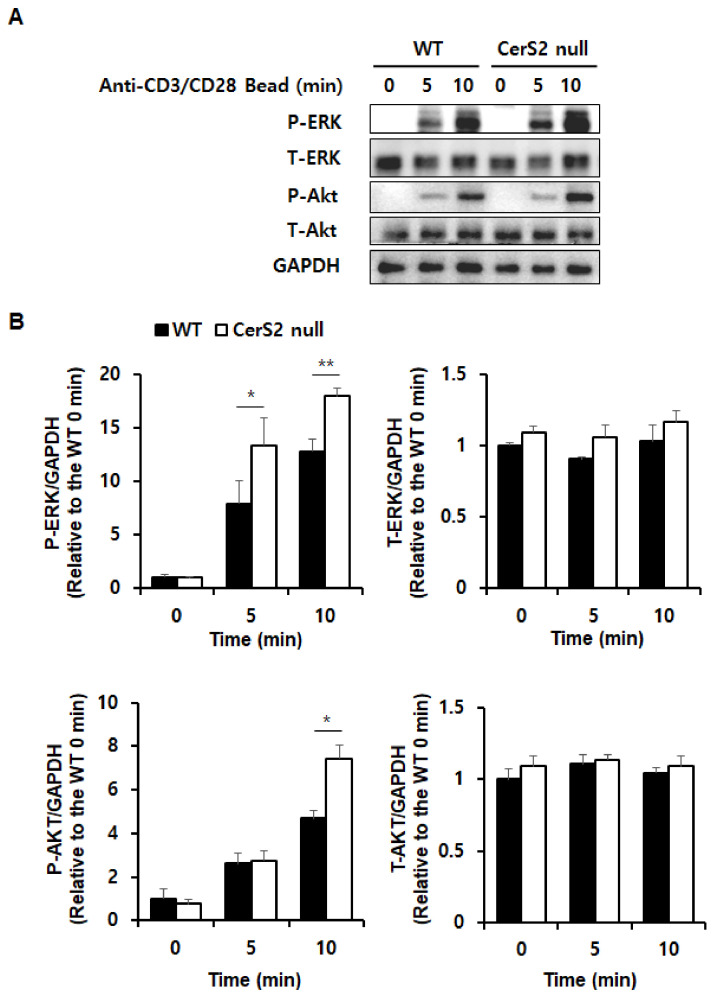
Wild type (WT)-CD4+ T cells and CerS2 null-CD4+ T cells differ in T cell receptor signal strength during T cell receptor stimulation. (**A**) Splenic CD4+ T cells from WT and CerS2 null mice were stimulated with anti-CD3/CD28 beads for 5 and 10 min, followed by western blotting to detect phosphorylated ERK, total ERK, phosphorylated AKT, and total AKT. Glyceraldehyde 3-phosphate dehydrogenase (GAPDH) was used as an internal control. (**B**) The pixel density of each protein band was divided by that of GAPDH for normalization. Data are expressed as the mean ± SEM (*n* = 3). * *p* < 0.05; ** *p* < 0.01. The representative images are shown of three independent experiments. Statistical significance was analyzed using the two-way analysis of variance.

SLs are components of the biological plasma membrane. Since the plasma membrane is the location where the TCR and co-molecules are recruited and initiate signals, we speculated that CerS2 may alter TCR signal strength. Strong TCR signals have been shown to prevent default Th2 programs, rather than actively driving Th1 polarization [29]. Because we confirmed that CerS2-null CD4+ T cells weakly promoted Th2 response, both in vivo and in vitro, we investigated the activation of TCR signal proteins, such as extracellular-regulated kinase (ERK) and AKT by performing western blotting. In short term stimulation with anti-CD3 and anti-CD28 antibodies, phosphorylation of ERK was significantly increased in CerS2 null-CD4+ T cells, while the expressions of total ERK were similar between WT and CerS2 null-CD4+ T cells (Figure 6A,B). In addition, CerS2 null-CD4+ T cells showed increased expression of phosphorylated AKT, when compared to WT-CD4+ T cells (Figure 6A,B). There was no significant difference in the expression of total AKT (Figure 6A,B). To confirm the higher TCR signal strength of CerS2 null-CD4+ T cells, we also examined signal strength upon stimulation with anti-CD3 alone (Figure S3). CerS2 null-CD4+ T cells showed increased phosphorylation of ERK and AKT compared to WT-CD4+ T cells upon activation with anti-CD3 alone, and no significant difference in the expression of total ERK and AKT was observed (Figure S3A,B).

## 3. Discussion

The possible role of ceramide in asthma has recently been studied. ORMDL sphingolipid biosynthesis regulator 3 locus is strongly associated with the risk of asthma [30], and ORMDL sphingolipid biosynthesis regulator 3 inhibits serine-palmitoyltransferase, the first and rate-limiting step of de novo SL synthesis [30,31,32]. Patients with uncontrolled asthma showed higher levels of C16:0 and C24:0 ceramides [33], and prevention of increases in lung ceramide levels mitigated allergen-induced apoptosis, reactive oxygen species, and neutrophil infiltration in mice [34]. Still, the exact mechanism of how ceramide contributes to the pathophysiological mechanism of asthma remains to be elucidated. CerS2 mainly synthesizes C22–C24 ceramides, and thus CerS2 null mice display a loss of very-long chain SLs [27]. Therefore, mild asthma symptoms in CerS2 null mice may suggest a pathological role of very-long chain SLs in asthma. Given the pivotal role that Th2 cells and cytokines play in asthma pathophysiology [35], the severely diminished Th2 responses observed in CerS2 null CD4+ T cells are likely to contribute to the decreased severity of asthma patients. 

According to a previous report [36], reduced invariant natural killer T (iNKT) cells were observed in the thymus, spleens, blood, and livers of CerS2-null mice, which rendered CerS2-null mice more susceptible to infection with a hepatotropic strain of the lymphocytic choriomeningitis virus [36]. The arrest of CerS2 null iNKT cell maturation in their development at the immature double positive stage may result from the lack of self-antigens, which are very-long chain glycosphingolipids, presented by CD1d molecules [36]. Very-long chain glycosphingolipids are major endogenous ligands required for iNKT cell maturation, survival, and maintenance, and levels of very-long chain ceramide and very long chain-glucosylceramide were reduced in CerS2 null thymocytes [36,37]. Thus, iNKT cells were markedly reduced in CerS2 null mice due to the lack of very-long chain glycosphingolipids [36]. T cells differentiate into functionally distinct effector subsets in response to exogenous stimuli, and innate immune cells including iNKT cells guide this process [38]. iNKT cells can affect the Th cell response. Activation of iNKT cells by α-galactosylceramide enhances the Th2 inflammatory response in the OVA-induced asthma model [39]. The Th2 inflammatory response and severity of asthma were both reduced in CD1d^−/−^ mice, which lack iNKT cells due to absence of the non-classical class I restricting element for iNKT cells, when sensitized and challenged with OVA [39]. In addition, iNKT cells play a pivotal role in limiting development of the Th17 lineage [38]. An augmented Th17 response was observed in mice lacking iNKT cells by comparing IL-17 production from antigen-experienced T cells from unmanipulated WT mice and iNKT-cell-deficient mice [38]. The mechanism used by iNKT cells to abrogate Th17 commitment by naïve CD4+ T cells required IL-4, IL-10, and IFN-γ [38]. Therefore, the decreased Th2 and enhanced Th17 responses observed in CerS2 null mice might be recapitulation of iNKT cell reduction. However, difference between CerS2 null mice and iNKT cell deficient mice exists. iNKT cells have been reported to affect the proliferation and response of memory, but not naïve, CD4+ T cells [40]. Similarly, Th2 differentiation from naïve CD4+ T cells was not altered in signaling lymphocytic activation molecule-associated protein (SAP) knockout mice, which exhibited severely impaired NKT cell development and virtually no iNKT cells [41,42]. However, in CerS2 null mice, Th2 differentiation from naïve CD4+ T cells was decreased, while Th17 differentiation from naïve CD4+ T cells was increased. Thus, alteration of Th2 and Th17 differentiation from naïve CD4+ T cells isolated from CerS2 null spleen cannot be solely explained by a deficiency of iNKT cells.

Notably, Th17 responses in total CD4+ T cells isolated from CerS2 null spleens were only higher upon stimulation with anti-CD3/anti-CD28, but not with PMA/ionomycin. Stimulation with anti-CD3/anti-CD28 activates CD4+ T cells via the TCR complex. By comparison, the combination of PMA, which is a protein kinase C activator structurally related to 1,2-diacylglycerol, and ionomycin, a calcium ionophore, bypass the TCR complex and activate several intracellular signaling pathways [43,44]. Besides iNKT deficiency, intrinsic alteration in the TCR response may be combined in CerS2 null mice. The TCR resides in ordered plasma membrane domains, which are also called lipid rafts, and aggregates upon TCR binding [45]. Altered membrane properties, including lipid rafts, have been previously reported in CerS2 null liver [46]. In addition, the biophysical properties of lipid extracts isolated from CerS2 null microsomes were different from those of WT mice, showing higher membrane fluidity and morphological changes [27]. CerS2 and its products, very-long-acyl chain SLs, are therefore considered important for membrane properties. Considering recent reports showing that plasma lipid composition and order can alter the functional phenotype of T cells including Th cell subsets, membrane changes by ablation of very-long-acyl chain SLs might affect TCR signaling [47]. Formation of TCR oligomers at the cell surface is termed TCR nanoclusters, and recent study showed the inhibitory role of membrane ceramide in TCR nanoclustering [48]. In addition, increased CerS2 expression was correlated with the impaired TCR nanoclustering, and CerS2 silencing restored TCR nanoclustering and activation in OT-II CCR5 knockout CD4+ T cells [48]. Therefore, the higher TCR strength observed in CerS2 null CD4+ T cells may also be derived from higher TCR nanoclustering induced by reduction of very-long chain ceramides in membrane.

The Gata3 and IL-4 mRNA increase induced by stimulation with anti-CD3 and anti-CD28 antibodies was significantly lower in CerS2 null-CD4+T cells, which implicates transcriptional regulation of IL-4. According to the previous study [49], in which the inhibitory activity of IL-4 production in activated T cells was investigated by ceramide derivatives, several ceramide derivatives significantly inhibited IL-4 production via transcriptional regulation in both PMA-activated T cells and antigen-primed cells. Because C16 ceramide is elevated in CerS2 null mice [27], increased C16 ceramide would decrease IL-4 transcription directly. However, C16 ceramide treatment did not inhibit IL-4 production in total CD4+ T cells isolated from murine WT spleen (data not shown). Decreased IL-4 transcription was also reported in iNKT cell deficient mice [50]. Similarly, the impaired IL-4 production by SAP-deficient T cells was associated with reduced Gata3 induction following TCR stimulation [51]. Therefore, decreased IL-4 transcription observed in CerS2 null CD4+ T cells may also be attributed to iNKT cell deficiency. In contrast to a consistent relationship between IL-4 production and Gata3 levels, RORγt was not altered despite higher IL-17A production in CerS2 null CD4+ T cells. Higher TCR strength in CerS2 null CD4+ T cells could be the possible mechanism about inconsistency between IL-17A and RORγt levels, since TCR signaling can affect IL-17A production regardless of RORγT or RORα levels [52].

Among six mammalian CerS, CerS2 mRNA expression was dominant in CD4+ T cells, and significant elevation of CerS2 expression was observed upon anti-CD3/anti-CD28 stimulation, implying a possible role of CerS2 in TCR activation. Indeed, higher ERK and AKT signaling in CerS2 null CD4+ T cells was observed upon anti-CD3 stimulation, which implied higher TCR signal strength. TCR strength has been considered as an important regulator of naïve CD4+ T cell differentiation [53]. For example, strong TCR stimulation in naïve CD4+ T cells favors Th1 differentiation over Th2 differentiation, both in vitro and in vivo [53,54,55]. In contrast, weak TCR strength in naïve CD4+ T cells tends to result in Th2 differentiation [54,55]. Thus, higher TCR responses in CerS2 null CD4+ T cells may contribute to lower Th2 differentiation. Although the effect of TCR signaling strength on Th17 differentiation still remains controversial [53], expression of IL-17A is particularly sensitive to the strength of TCR signaling, requiring full activation of Ca^2+^-mediated pathways, in addition to signals from cytokines required for the induction and activation of RORγT and STAT3 [52]. CD4+ T cells lacking Itk, a tyrosine kinase required for full TCR-induced phospholipase C-γ activation, displayed diminished IL-17A expression, despite unaltered expression of RORγT [52]. In addition, optimal expression of IL-17A requires high doses of anti-CD3/anti-CD28 or high dose antigen stimulation [52]. In the present study, a higher Th17 response with anti-CD3/anti-CD28 stimulation was observed in CerS2 null CD4+ T cells without alteration of RORγT levels, and a higher Th17 response in CerS2 null CD4+ T cells was only observed upon stimulation with anti-CD3/anti-CD28, but not with PMA/ionomycin. Higher TCR responses in CerS2 null CD4+ T cells may therefore lead to optimal expression of IL-17A and a higher Th17 response. According to the previous reports [52,56,57], low TCR signal strength favors differentiation into regulatory T cells (Treg), even in the presence of Th17-inducing factors, whereas Th17 differentiation requires high TCR signals. Furthermore, the concentration of anti-CD3 antibody positively correlates with the percentage of cells induced to express IL-17A upon Th17 differentiation, which depends on activity of nuclear factor of activated T cells [52,57]. Since strong activation of the Akt/mTOR pathway induced by high TCR signal strength is negatively correlated with Treg cell differentiation, high TCR signal strength can induce Th17-skewed immune response.

In contrast to CerS2 deficiency, CerS6 knockout T cells showed reduced levels of tyrosine phosphorylation upon stimulation with anti-CD3, suggesting that TCR signaling was impaired in the absence of CerS6 [26]. In contrast to the normal proliferation of CerS2 null CD4+ T cells, CerS4 knockout CD4+ T cells showed faster proliferation rates, and CerS6 knockout CD4+ T cells exhibited reduced ability to proliferate compared with WT counterparts [26]. These results therefore confirmed the distinct role of each CerS and its metabolites, SLs with specific-acyl chain lengths, in the functioning of CD4+ T cells. Ceramides have been suggested as potential biomarkers of allergic asthma, and intratracheal ceramide administration resulted in lung inflammation, tissue remodeling, and airway flow obstruction [58,59]. Moreover, nasal delivery of the drugs, which reduce ceramide levels, mitigated airway inflammation and mucus production in asthma models [60]. The current study showed that CerS2 deficiency caused reduced Th2 response and alleviated OVA-induced asthma, and suggests an important role of CerS2 and its products in asthma pathogenesis. The present study implicates that altered SL acyl chain length may contribute to the pathophysiological mechanism of CD4+ T cell-mediated inflammatory diseases such as asthma.

## 4. Materials and Methods

### 4.1. Animal Experiments

All procedures were approved by the Animal Care and Use Committee of Ewha Woman’s University, School of Medicine (ESM17-0389, date of approval 14 November 2017). CerS2 null mice were described previously [27,61]. Eight to 10-week-old female mice were housed under specific pathogen-free conditions on a 12-h light and dark cycle with free food and water access. Mice were immunized by intraperitoneal injection with 10 μg OVA (Sigma-Aldrich, St. Louis, MO, USA) complexed with 1 mg aluminum hydroxide (Sigma-Aldrich) in a volume of 200 μL per mouse on day 0 and day 5. For post-immunization, mice were exposed on day 12 to 15 for 30 min per day to an aerosol of either 1% OVA in PBS, or PBS only using a nebulizer. Twenty-four hours after final aerosol challenge, the mice were euthanized to collect BAL fluid and lung tissues.

### 4.2. BAL

After mice were sacrificed with a mixture of Zoletil (30 mg/kg, Virbac Laboratories, Carros, France) and Rompun (10 mg/kg, Bayer, Leverkusen, Germany), the trachea was cannulated using a venipuncture needle. BAL isolation was subsequently conducted via the instillation of 0.5 mL PBS into the lungs, along with gentle massaging to maximize cell recovery. BAL cells were pelleted via centrifugation and counted using a hemocytometer.

### 4.3. Histological Evaluation

Lung tissues were fixed in 4% paraformaldehyde, embedded in paraffin, and then sectioned at a thickness of 4 μm. The sections from the paraffin block were deparaffinized and stained with hematoxylin and eosin, according to standard methods for histological evaluation.

### 4.4. Isolation of Total CD4+ T Cells and Their Activation

Total CD4+ T cells were purified from spleens of WT and CerS2 null mice using the MACS system (Miltenyi Biotec, Gladbach, Germany). Cells were activated with either plate-bound anti-CD3 and soluble anti-CD28 or PMA (32 nm) and ionomycin (1.34 nm) for 3 days and 16 h, respectively. For detection of TCR signaling, cells were stimulated with anti-CD3/CD28 beads (Invitrogen, Oslo, Norway) or anti-CD3 (10 μm alone for indicated time.

### 4.5. Isolation of Naïve CD4+ T Cells and T Helper Cell Differentiation

Naïve CD4+ T cells were isolated from WT and CerS2 null spleen using magnetic beads, using the naïve CD4+ T cell isolation kit for mice (Miltenyi Biotec) according to manufacturer’s instructions. Isolated cells were primed with plate-bound anti-CD3 (5 μg/mL; BD Biosciences, San Jose, CA, USA) and soluble anti-CD28 (1 μg/mL; BD Biosciences) in round bottomed 96-well plates with RPMI1640 medium containing 10% fetal bovine serum and 1% penicillin/streptomycin (HyClone, Logan, UT, USA). For Th1 differentiation, the cells were cultured with recombinant murine IL-2 (20 ng/mL, R&D Systems, Minneapolis, MN, USA), recombinant murine IL-12 (10 ng/mL, PeproTech, Rocky Hill, NJ, USA) and 2 μg/mL anti-mouse IL-4 (2 μg/mL, R&D systems) for 48 h. For Th2 differentiation, the cells were stimulated with recombinant murine IL-2 (20 ng/mL; R&D Systems), recombinant murine IL-4 (10 ng/mL; BioLegend, San Diego, CA, USA), anti-mouse IFN-γ (2 μg/mL; R&D Systems), and anti-mouse IL-12 (2 μg/mL; R&D Systems). For Th17 differentiation, the cells were cultured with recombinant murine IL-2 (20 ng/mL; R&D Systems), recombinant murine TGF-β (5 ng/mL; BioLegend), recombinant murine IL-6 (25 ng/mL; PeproTech), anti-mouse IFN-γ (2 μg/mL; R&D Systems), and anti-mouse IL-4 (2 μg/mL; R&D Systems).

### 4.6. ELISA

ELISA was performed to determine IFN-γ, IL-4, and IL-17A levels in cell culture media of CD4+ T cells using commercial kits from BioLegend.

### 4.7. Real-Time Polymerase Chain Reaction

Total mRNA was isolated from activated CD4+ T cells using the RNeasy Mini Kit (Qiagen, Valencia, CA, USA). Complementary DNA was synthesized from the mRNA using a Verso cDNA synthesis kit (Thermo Fisher Scientific, Waltham, MA, USA) according to the manufacturer’s protocols. Quantitative PCR was performed using the ABI PRISM 7500 fast sequence detection system (Applied Biosystems, Foster City, CA, USA). Gene expression was normalized to the hypoxanthine phosphoribosyltransferase reference gene. Relative gene expression was determined as 2^−ΔΔCT^, as previously described [62]. The primers were as follows: mouse hypoxanthine phosphoribosyltransferase, TaqMan™ (Mm00446968_m1); mouse CerS1, TaqMan™ (Mm00433562_m1); mouse CerS2, TaqMan ™ (Mm00504086_m1); mouse CerS3, TaqMan™ (Mm03990709_m1); mouse CerS4, TaqMan™ (Mm01212479_m1); mouse CerS5, TaqMan™ (Mm00510996_g1); mouse CerS6, TaqMan™ (Mm00556165_m1); mouse Gata3, forward 5′-CAG CTG CCA GAT AGC ATG AA-3′ and reverse 5′-CAT AGG GCG GAT AGG TGG TA-3′; mouse RORγt, forward 5′-GGA GCT CTG CCA GAA TGA GC-3′ and reverse 5′-CAA GGC TCG AAA CAG CTC CAC-3′ [63]. TaqMan primers were purchased from Applied Biosystems.

### 4.8. Western Blotting

Cultured CD4+ T cells were lysed in radioimmunoprecipitation assay buffer (50 mM Tris-Cl, pH 7.5), 150 mM NaCl, 1% Nonidet P-40, 0.5% sodium deoxycholate, and 0.1% sodium dodecyl sulfate) containing protease and phosphatase inhibitors (Sigma-Aldrich). Protein samples were resolved using sodium dodecyl sulfate-polyacrylamide gel electrophoresis and then transferred to nitrocellulose membranes. After blocking with 5% bovine serum albumin for 1 h, the membranes were incubated with primary antibodies overnight at 4 °C, followed by peroxidase-conjugated secondary antibodies (Jackson ImmunoResearch, West Grove, PA, USA) for 1 h at room temperature. The bands were detected by Image Quant LAS-4000 (GE Healthcare Life Sciences, Fuji, Japan) using ECL Western blotting detection reagents (Amersham Biosciences, Little Chalfont, UK). The band pixel densities of proteins were divided by the pixel densities of the corresponding glyceraldehyde 3-phosphate dehydrogenase (GAPDH) bands for quantitation using UN-SCAN-IT-gel 6.1 software (Silk Scientific, Orem, UT, USA).

### 4.9. Measurement of CD4+ T Cell Proliferation

Isolated CD4+ T cells were labeled with carboxyfluorescein diacetate succinimidyl ester (CFSE, CellTrace™ CFSE Cell Proliferation kit; Thermo Fisher Scientific) in accordance with the manufacturer’s instructions. After CFSE labeling, CD4+ T cells were incubated for 5 days in the presence of anti-CD3 and anti-CD28 antibodies, followed by flow cytometry for comparing degrees of proliferation. To examine the proliferation capacity of CD4+ T cells upon OVA stimulation, mice were immunized by intraperitoneal injection with 100 μg OVA (Sigma-Aldrich) complexed with 1 mg aluminum hydroxide (Sigma-Aldrich) in a volume of 200 μL per mouse on day 0 and day 5. After 7 days, mice were sacrificed and total CD4+ T-cells were isolated from spleens of WT and CerS2 null mice using MACS system (Miltenyi Biotec) for CFSE labeling. CD4- cells were seeded onto a culture dish and incubated for 4 h to collect the adherent cells by gentle pipetting, and these cells were used as antigen presenting cells. Antigen presenting cells pre-treated with 50 ng/mL of PMA and OVA (4 mg/mL) for 1 h were washed and co-cultured with CFSE-labeled CD4+ T cells in the presence or absence of OVA (100 μg/mL) for 5 days. To support survival of T cells, IL-2 (20 ng/mL) was added to the wells, and after 5 days, the degree of proliferation of CFSE labeled T cells was compared by flow cytometry.

### 4.10. Flow Cytometry

To analyze CD4+ T cell profiles in BAL fluid, surface expression of CD4 and intracellular expressions of IFN-γ, IL-4, and IL-17 on BAL cells were detected by flow cytometry. BAL cells were stained with the following antibodies: fluorescein isothiocyanate anti-mouse CD4 (BD Biosciences), allophycocyanin anti-mouse IFN-γ (BD Biosciences), allophycocyanin anti-mouse IL-4 (BioLegend), and PerCP/Cy5.5 anti-mouse IL-17A (BD Biosciences). The BAL cells were then analyzed using a NovoCyte 3000 Flow Cytometer (ACEA Biosciences Santa Clara, CA, USA).

### 4.11. Statistical Analyses

The data are presented as the mean ± SEM. Statistical significance was analyzed using the two-way analysis of variance. A value of *p* < 0.05 was considered statistically significant.

## 5. Conclusions

The present study suggested that alterations of SL acyl chain length could modulate the TCR strength of CD4+ T cells, which affected the Th response and differentiation. Modulation of the Th response of CD4+ T cells by altered SL acyl chain length may contribute to the pathophysiological mechanism of various immune-mediated human diseases, in which CD4+ T cells are involved. Finally, CerS2 and very-long acyl chain SLs may in the future be used as therapeutic targets for the treatments of asthma and Th2-related diseases.

## Supplementary Materials

The following are available online at https://www.mdpi.com/1422-0067/22/5/2713/s1.

## Abbreviations

BAL	Bronchoalveolar lavage
CD4	Cluster of differentiation 4
CerS	Ceramide synthase
CFSE	Carboxyfluorescein diacetate succinimidyl ester
ELISA	Enzyme-linked immunosorbent assay
Erk	Extracellular-regulated kinase
GAPDH	Glyceraldehyde 3-phosphate dehydrogenase
IFN-γ	Interferon-γ
IL	Interleukin
OVA	Ovalbumin
RORγt	RAR-related orphan receptor gamma
SAP	Signaling lymphocytic activation molecule-associated protein
SL	Sphingolipid
TCR	T-cell receptor
Th	T-helper
Treg	Regulatory T cells
WT	Wild type
