# Supplementary material for: Ceramide Synthase 2 Null Mice Are Protected from Ovalbumin-Induced Asthma with Higher T Cell Receptor Signal Strength in CD4+ T Cells
Source: Int J Mol Sci. 2021 Mar 8;22(5):2713. doi: 10.3390/ijms22052713 (PMC7962461; doi:10.3390/ijms22052713)
Supplement: Supplementary file 1 [file ijms-22-02713-s001.pdf]

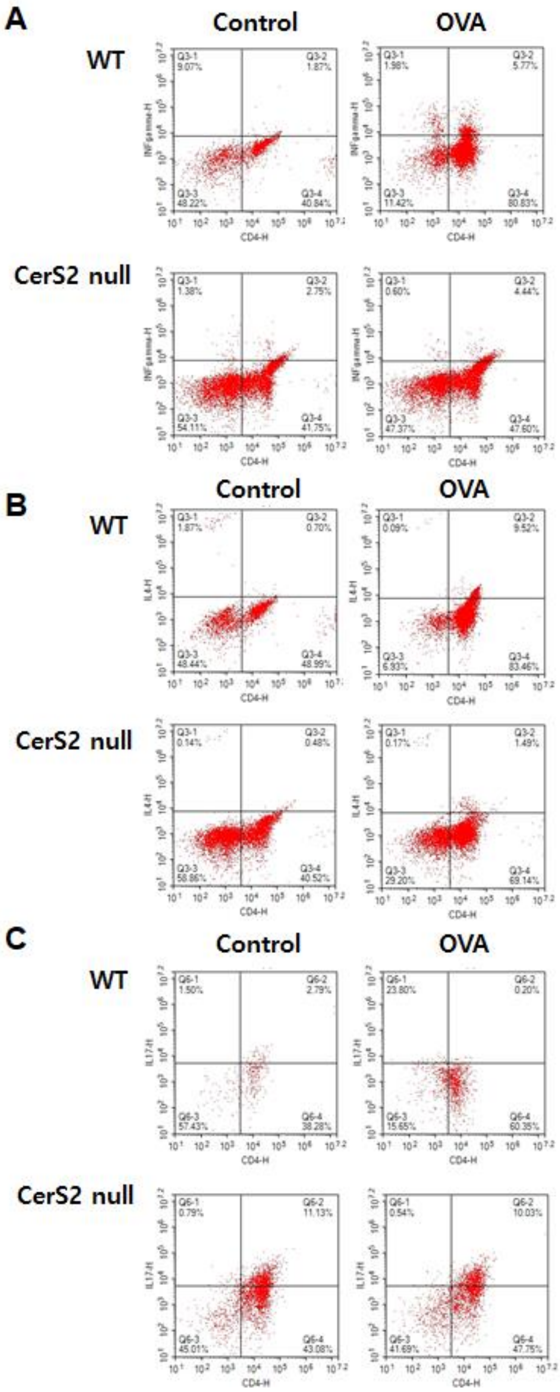

Figure S1. In ovalbumin-induced allergic airway inflammation, WT and CerS2 null mice exhibit different helper T cell profiles in CD4+ cells in bronchoalveolar lavage cells. Flow cytometric analyses were performed to compare helper T (Th) cell profiles of CD4+ T cells. The representative flow cytometric data of (F) CD4 + IFN-γ + Th1 cells, (G) CD4 + IL-4 + Th2 cells, and (H) CD4 + IL-17 + Th17 cells were shown. The representative images are shown of three independent experiments. CerS2, Ceramide synthase 2; OVA, Ovalbumin, WT, Wild-type.

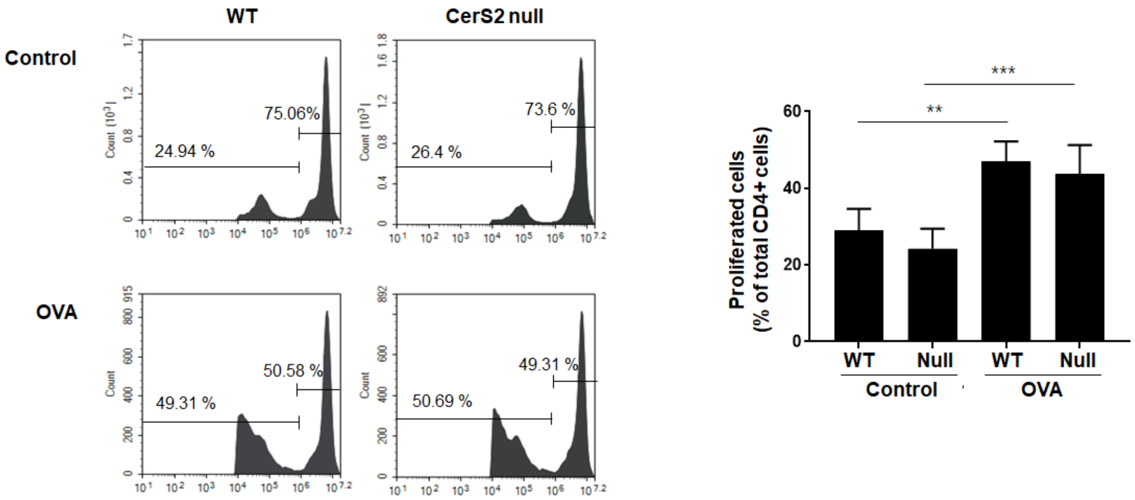

Figure S2. The extent of proliferation of CerS2 null-CD4+ T cells is similar to that of WT upon ovalbumin (OVA) stimulation. To examine the proliferation capacity of CD4+ T cells upon OVA stimulation, mice were immunized by intraperitoneal injection with 100 µg OVA complexed with 1mg aluminum hydroxide. CFSE-labelled CD4+ T cells from wild-type and CerS2 null mice were cultured with or without OVA (100 µg/mL) for 5 days, and the degree of proliferation was measured using flow cytometry (left). The percentages of proliferating cells was analysed (right). Data are expressed as means ± SEM (***P*< 0.01, ****P*< 0.001). Statistical significance was analysed using the two-way analysis of variance.

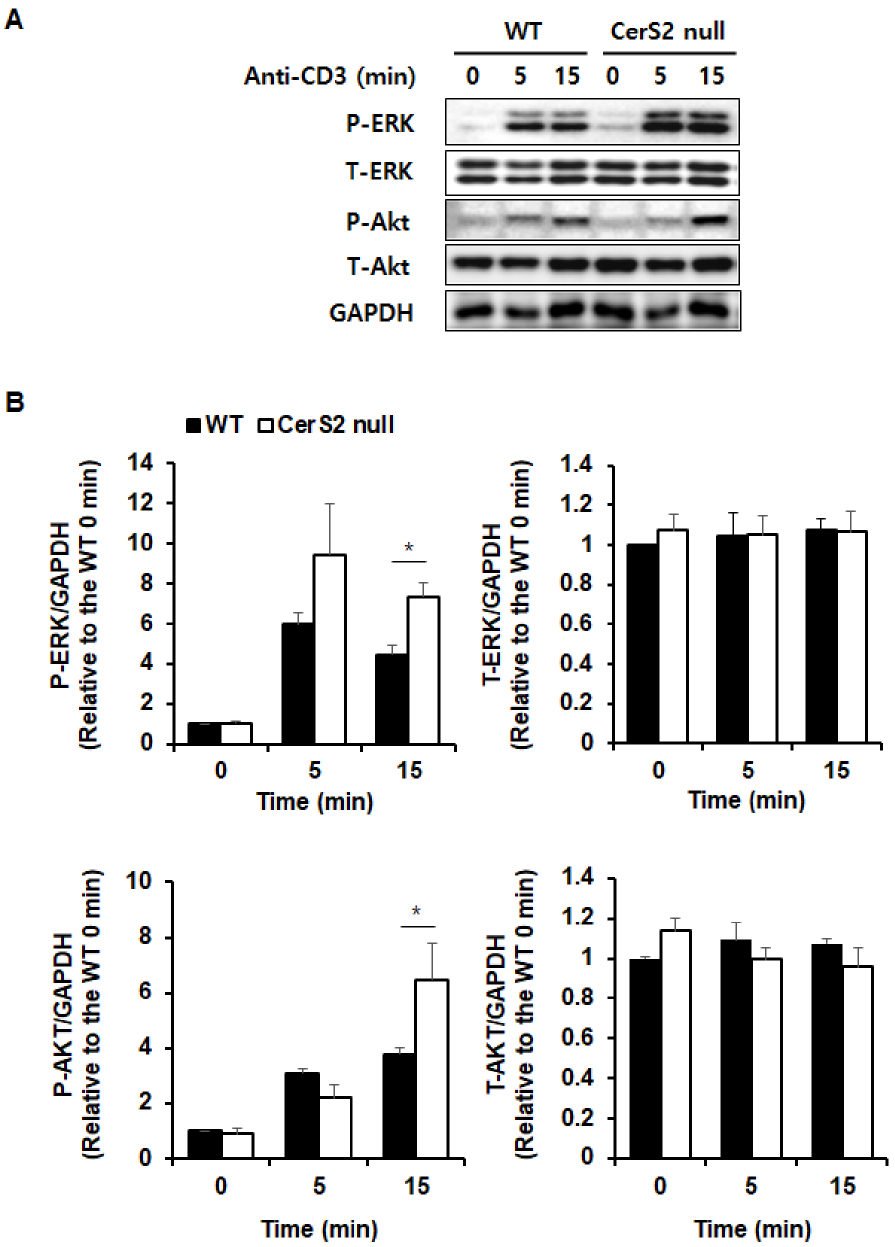

Figure S3. CerS2 null-CD4+ T cells display higher T cell receptor signal strength upon stimulation with anti-CD3 antibodies. (A) Splenic CD4+ T cells from WT and CerS2 null mice were stimulated with anti-CD3 antibodies for 5 and 15 min, followed by western blotting to detect phosphorylated ERK, total ERK, phosphorylated AKT, and total AKT. Glyceraldehyde 3-phosphate dehydrogenase (GAPDH) was used as an internal control. (B) The pixel density of each protein band was divided by that of GAPDH for normalization. Data are expressed as the mean ± SEM (n=3). $^{*}P < 0.05$; $^{**}P < 0.01$. The representative images are shown of three independent experiments. Statistical significance was analysed using the two-way analysis of variance.
